# The effects of the combined exercise intervention based on internet and social media software (CEIBISMS) on quality of life, muscle strength and cardiorespiratory capacity in Chinese postoperative breast cancer patients:a randomized controlled trial

**DOI:** 10.1186/s12955-019-1183-0

**Published:** 2019-06-26

**Authors:** Xiaosheng Dong, Xiangren Yi, Dezong Gao, Zan Gao, Shuyuan Huang, Mengyao Chao, Wenxin Chen, Meng Ding

**Affiliations:** 1 College of Physical Education, Shandong Normal University, 88 Wenhuaxi Road, Jinan, 250014 China; 2 College of Physical Education, Shandong University, Jinan, 250011 China; 3 The Department of Breast Surgery, The Second Hospital of Shandong University, Jinan, 250033 China; 4 School of Kinesiology, University of Minnesota, Minneapolis, MN 55455 USA

**Keywords:** Combined exercise breast cancer quality of life muscle strength cardiorespiratory capacity

## Abstract

**Background:**

Breast cancer (BC) patients who undergo surgery followed by radiotherapy and chemotherapy have limitations on physical activity which will lead to a decreased quality of life and poor physical fitness level. The purpose of this study was to investigate the effects of the combined exercise intervention based on internet and social media software (CEIBISMS) on postoperative breast cancer patients by evaluating their quality of life, muscle strength and cardiorespiratory capacity.

**Methods:**

This study was a randomized control trial with an intervention period of 12 weeks. Sixty participants (30 in each group, 42–60 years old, female) were recruited through an outpatient department. Procedure of exercise in the intervention group included: via phone step-recording app, ask the individuals to complete the target number of steps within a specified period of exercise, four times per week; face-to-face remote video instruction of individuals on muscle training, three times per week; via social media apps daily push common knowledge of physical exercise BC rehabilitation. The control group received traditional treatment and rehabilitation according to daily specifications of the hospital. The primary outcome was quality of life and the secondary outcomes were muscle strength and cardiorespiratory capacity.

**Results:**

Experiments using a Short Form 36 showed that the CEIBISMS yielded significantly better results than traditional methods, in vitality (*p* = 0.009), mental health (*p* = 0.001) and reported health transition (*p* = 0.048) by week 12. The CEIBISMS resulted in significant improvement in the stand-up and sit-down chair test (*p* < 0.0001), arm lifting test (*p* = 0.017).

**Conclusion:**

The CEIBISMS offered rehabilitative effects in quality of life (QOL) and muscle strength of postoperative patients with breast cancer (BC) in China.

**Trial registration:**

ChiCTR-IPR-17012368. Trial registered on 14 August, 2017.

## Introduction

Breast cancer (BC) is a malignant cancer that severely harms women’s health worldwide and is the most common cancer in women in both developed and developing countries. In developing countries, the incidence of BC is rising due to increased life expectancy, the expansion of urbanization, and the adoption of a western lifestyle [1]. The most common manifestations in postoperative BC patients are mental fatigue, anxiety, and depression [2–4]. Moreover, the physical inactivity due to weakness and upper extremity function limitation after surgery may cause the decrease in muscle strength and cardiorespiratory endurance, and then seriously affect the mood and QOL of BC patients [5, 6].

Some results of previous studies demonstrated that exercises may improve muscle strength and cardiorespiratory endurance in BC patients during rehabilitation and further improve their emotions and QOL [5, 7–12]. However, there are some problems in the current exercise rehabilitation forms. First, patients are usually unable to adhere to regular group exercise rehabilitation in hospital outpatient clinics or fitness centers due to the traffic delays or personal schedules; second, although face-to-face private exercise guidance may have a better rehabilitation effect, it will increase the economic burden of patients.

With the universalization and extensive applications of the internet, exercise intervention and guidance for BC patients via phone social media apps and remote video have become possible. Related studies have been carried out [13–15]. However such studies have some deficiencies such as a singular method of exercise intervention and exercise guidance [16–19]. Therefore, due to the limitations of previous studies, we aimed to investigate the effects of the CEIBISMS on QOL, muscle strength and cardiorespiratory capacity in postoperative BC patients in China.

## Methods

### Study design and participants

The present study was a randomized single-blinded controlled trial to investigate the effects of the CEIBISMS in postoperative BC patients.

Participants were recruited from the Department of Breast Surgery, The Second Hospital of Shandong University, Jinan City, Shandong Province. All participants should sign the written consents before participating in the study.

Inclusion criteria: Patients with BC at phase I to III who have finished postoperative radiotherapy/chemotherapy within 4 months to 2 years.

Exclusion criteria: Patients who 1) have communication or language barrier and cannot complete the questionnaire; 2) have metastasis, severe deterioration of conditions or severe mental disorder; 3) have a history of acute suicidality; 4) have cognitive brain organic lesion and dementia; 5) can not use smartphone apps or tele-video. Patients who met one or more of the above criteria would be excluded from the study.

### Intervention

#### Control group

Subjects randomized into the control group received traditional treatment and rehabilitation according to daily specifications of the hospital. The traditional treatment and rehabilitation were consistent with the recommendations of the National Institute for Health and Care Excellence clinical guidance (NG101) [20]. The rehabilitation was also performed according to regular requirements and postoperative BC rehabilitation-related health education was provided.

#### Intervention group

Basic contents of intervention in the intervention group included muscle training, cardiopneumatic endurance training and postoperative rehabilitation knowledge which pushed diverse interventions. (1) Muscle training: muscle training includes muscle strength, muscle endurance and muscle function training. Face-to-face televideo instruction on physical exercise rehabilitation was performed 3 times per week in 30-min sessions, including 5 min warm-up, 20 min muscle training and 5 min relaxation each time. The primary content of muscle training in the first month was endurance training, in the second month was strength training, and in the third month was muscle function training [21]. (2) Cardiorespiratory capacity training: face-to-face televideo instruction was performed 4 times per week, exercise strength was determined via Ratings of perceived exertion (RPE), subjects were asked to complete the target number of steps within a specified time, number of steps were recorded via phone step-recording app; (3) Postoperative BC rehabilitation knowledge: common knowledge of physical exercise BC rehabilitation was pushed regularly through social media apps every day; knowledge of simple physical exercise rehabilitation and psychological adjustment relative to BC survivors was also pushed.

Physical exercise rehabilitation training was performed under the guidance of professional physiotherapists of the subjects. The measures of exercise intervention were gradually advanced and the strength of exercise was gradually increased. The side effects and severe adverse events would be recorded and reported to the study designer by physiotherapists, and the study designer determined further measures to take. If necessary the training would be discontinued according to the reports from the therapists and results of consultation with BC specialists.

## Variables

### Outcomes measured at baseline

#### Clinical history and socio-demographic characteristics

Information on age, gender, smoking, comorbidities (such as lymphedema, hyperlipidaemia, overweight, obesity, depression, anxiety and osteoporosis), height and weight was obtained from face-to-face interviews and measurement method.

### Outcomes measured at baseline and at the end of the CEIBISMS intervention programme

#### Primary outcomes

The primary outcome was QOL, which was determined by the 36-item Short Form Health Survey (SF-36) [22, 23]. SF-36 is a reliable and manageable questionnaire measuring QOL which has been extensively applied in assessment of the health conditions of patients with chronic diseases. SF-36 includes 8 dimensions and 2 summary tables, and the score of each dimension is in the range of 0 to 100 (100 represents the best health condition).

#### Secondary outcomes

The secondary outcomes were muscle strength measured with the stand-up and sit-down chair test and arm lifting test, and cardiorespiratory capacity measured with maximal oxygen uptake (VO2max).

Stand-up and sit-down chair test (number of times standing up from the chair within 30 s) [24]: This test is used to evaluate leg strength and endurance of the subject. Procedure of testing: 1) Put an upright chair (or a folding chair) against the wall (for the sake of safety). 2) The subject sits in the middle of the chair with right foot and left foot apart equal to the shoulders, and one foot may be put slightly front and the other slightly back. Both arms were crossed around the waist and close to the chest. 3) During the test, the subject should completely stand up and then completely sit down. 4) Record the number of times that the subject stands up from and sits down in the chair within 30 s. 5) For safety purposes or when necessary, the subject may use her arms for assistance.

Arm lifting test (30 s dumbbell of 5 pounds or 2.3 kg lifting test): This test is used to evaluate the strength and endurance of upper extremities. Procedure of testing [24]: 1) 2.3 kg (5 pounds) dumbbell is used. 2) Put an upright chair (or a folding chair) against the wall (for the sake of safety). 3) Sit close to the side of chair if the subject has a stronger arm on that side. 4) The non-affected arm holds the dumbbell with the arm naturally hanging straight down over one side of the chair while the body takes a push-up position. 5) The non-moving arm is fixed close to the body so that only the moving arm is moving. 6) First bend the arm into push-up position, then straighten to maximum extent while gradually rotating outward to a palm upward position. 7) Next, the arm returns to the push-up position and is put on one side of the chair. 8) Record the number of times that the arm bends and lifts. 9) Precautions: Make sure the arm is completely bent, and then completely straighten the elbow; it is very important that the upper extremities remain stable without swaying.

Maximal oxygen uptake: this outcome was determined by an modified Bruce treadmill protocol [21] which was strictly abided by, and the heart rate was monitored with a polar watch during the test. The protocol consists of two to four sessions of continuous exercise lasting for 3-min. The test was designed to raise the steady state HR of the subject to between 110 beats/min and 70% heart rate reserve (HRR) (or 85% of the age-predicted HRmax) for at least two consecutive stages. It was important to remember that two consecutive HR measurements must be obtained within this HR range to predict VO2max.

The measurements of all outcomes were recorded at baseline and 12 weeks after intervention. Measurements were conducted in the same place and by the same tester for both intervention group and control group strictly according to the precautions and operational rules, to ensure that the measurements were scientific and precise.

#### Sample size

Sample size was estimated based on our preliminary trial. In the preliminary trial of 16 patients, the vitality score were 54.7 ± 8.4 and 62.5 ± 12.3 in the control and intervention group, respectively, after 12 weeks intervention. According to the previous study, we calculated that thirty women were enrolled in each group for a 80% power to detect a 8 difference in vitality between the groups with SD of 10, α level of 0.05 and allowing for a 25% loss to follow-up.

#### Randomization allocation

The patients admitted to the study were randomized to either the intervention group or the control group. The randomization sequence was generated by the study designer using a random number table.

#### Blinding procedure

Allocation was retained by the study designer until the end of the study to ensure allocation concealment. Outcome assessors and data analysts were kept blinded to the allocation. Patients and treatment providers were advised not to reveal their group allocation to the outcome assessors.

### Data analysis

The study results underwent statistical analysis with SPSS software version 19.0 (SPSS Inc., Chicago, IL, USA). Analysis of covariance adopted for comparisons between groups, and paired *t* test was used for before-exercise and after-exercise comparisons within a group, whereas *p* < 0.05 represent there is a significant difference between the test results.

## Results

### Participant characteristics

**Fig. 1:**
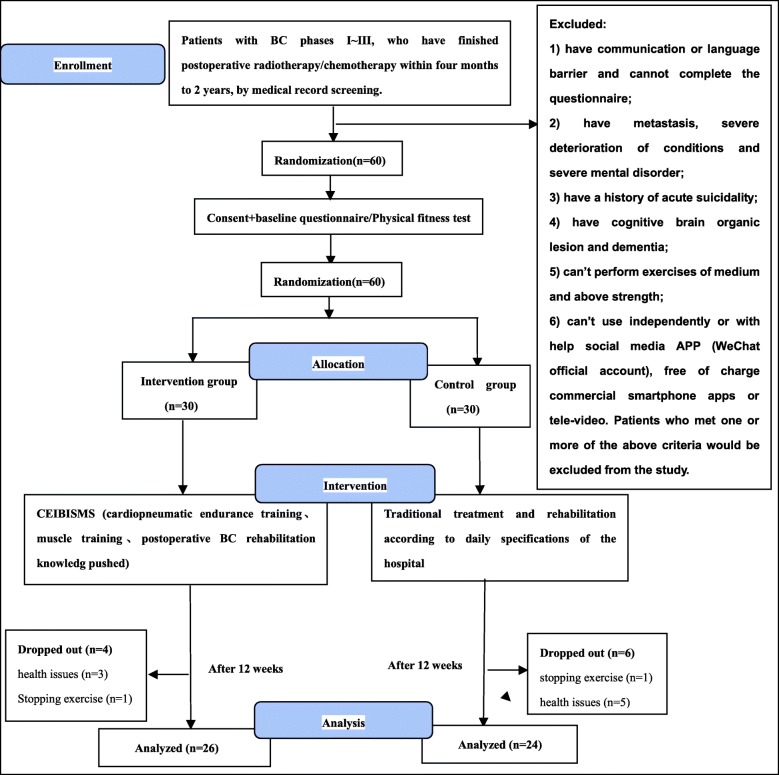
The flow chart of the trial design. Abbreviations: BC brest cancer, CEIBISMS combined exercise intervention based on internet and social media software

Sixty participants were randomly assigned to the groups: intervention group (*n* = 30, female), control group (*n* = 30, female). Over the course of the study, 4 participants in the intervention group withdrew because of health issues (n = 3) or stopping exercise (*n* = 1); 6 participants in the control group withdrew due to health issues (*n* = 2) and 4 participants were lost during the follow-up in the control group (*n* = 4). The data of the 50 participants who completed the trial were included in the final analysis. Figure 1 shows the process of subjects following the recommendation of the Consolidated Standards of Reporting Trials (CONSORT) [25].

The ranges of the age, body age and BMI of the subjects were 42–60 years old and 22–28 BMI, respectively. Table 1 shows the characteristics of subjects. No differences were found among the groups for the values of anthropometric (age = 0.056 and BMI = 0.114), marital status (*p* = 0.735), hemodynamics (BPS at rest *p* = 0.204, BPD at rest *p* = 0.567), stage of illness (*p* = 0.600), phase of treatment (*p* = 0.207) by using one way analysis of variance (ANOVA) analysis.

**Table 1:** Subject characteristics for each group. Data are means (±SD)

Demographic	Intervation group (*n* = 26)	Control group (*n* = 24)	Baseline ANOVA analysis (P)
Anthropometric			
Age (years)	48.00(5.54)	51.63(7.49)	0.056
BMI	24.07(1.98)	25.20(2.94)	0.114
Marital Status			0.735
Married	15(57.69%)	15(62.50%)	
Single	11(42.31%)	9(37.50%)	
Hemodynamics			
BPS at rest (mmHg)	118.73(14.38)	126.00(24.61)	0.204
BPD at rest (mmHg)	76.846(11.08)	75.08(10.47)	0.567
Stage of illness			0.600
I	6(23.08%)	10(41.67%)	
II	18(69.23%)	10(41.67%)	
III	2(7.69%)	4(16.67%)	
Phase of Treatment			0.207
Observation	2(7.69%)	4(16.67%)	
Chemotherapy	10(38.46%)	11(45.83%)	
Radiation therapy	0(0%)	0(0%)	
Radiochemotherapy	14(53.85%)	9(37.5%)	

Abbreviations: *ANOVA* Analysis of Variance, *KG* Kilogram, *BMI* body mass index

### Primary outcomes

#### The 36-item short form health survey (SF-36)

In the intervention group, participants showed significant improvements after 12 weeks in role-physical (*p* = 0.009), general health (*p* = 0.024), mental health (*p* = 0.014), vitality (*p* = 0.014) and reported health transition (*p* = 0.007). As well as marginally significant improvements in physical functioning (*p* = 0.085), the score was increased in bodily pain (*p* = 0.290), social functioning (*p* = 0.148) and role-emotional (*p* = 0.144) subscales. In the control group, participants had marginally significant improvement after 12 weeks in physical functioning (*p* = 0.079), general health (*p* = 0.054). But there was no significant difference. There were statistically significant differences among two groups in vitality (*p* = 0.009), mental health (*p* = 0.001) and reported health transition (*p* = 0.048) (Table 2).

**Table 2:** The means (±SD) of the Mos 36-item Short Form Health Survey, Stand-up and sit-down chair test, Arm lifting test and Maximal oxygen uptake in two groups

Variables	Intervention Group		Intervention Group Paired t test (P)	Control Group		Control Group Paired t test(P)	Baseline ANOVA analysis (P)	ANCOV Aanalysis(P)
	(*n* = 26)			(*n* = 24)				
	Baseline	12 weeks		Baseline	12 weeks			
SF-36								
PF	82.12(10.69)	86.73(7.87)	0.085	78.96(18.88)	85.21(10.78)	0.079	0.466	0.735
RP	32.69(38.58)	57.69(37.93)	O.009*	59.38(42.23)	53.13(41.25)	0.519	0.057	0.142
BP	73.46(13.84)	77.31(13.43)	0,290	73.33(18.10)	79.58(16.28)	0.189	0.978	0.590
GH	65.96(15.85)	73.38(18.16)	0.024*	57.21(19.80)	63.08(18.90)	0.054	0.090	0.300
VT	61.92(11.67)	67.12(5.86)	0.014*	62.71(8.84)	62.08(10.31)	0.700	0.791	0.009*
SF	88.94(17.08)	95.67(17.30)	0.148	85.42(24.91)	93.75(21.49)	0.103	0.560	0.872
RE	61.54(40.76)	74.36(33.08)	0.144	56.94(44.48)	69.44(40.43)	0.131	0.705	0.744
MH	51.08(6.23)	54.62(4.92)	0.014*	51.83(6.62)	49.83(5.53)	0.197	0.679	0.001*
HT	2.42(1.14)	1.65(0.94)	0.007*	2.46(1.28)	2.17(0.82)	0.373	0.918	0.048*
SPSDCT	14.81(2.93)	19.31(4.32)	0.000*	15.17(3.41)	15.67(3.69)	0.180	0.691	0.000*
ALT	16.08(5.01)	20.50(4.14)	0.000*	18.58(4.09)	19.33(4.46)	0.268	0.060	0.017*
VO2max	43.07(18.32)	50.58(13.88)	0.027*	41.35(17.71)	44.98(16.52)	0.056	0.738	0.149

Abbreviations: SF-36 the Mos 36-item Short Form Health Survey, PF Physical Functioning, RP Role-Physical, BP Bodily Pain, GH General Health, VT Vitality, SF Social Functioning, RE Role-Emotional, MH Mental Health, HT Reported Health Transition, VO2max maximal oxygen uptake, SPSDCT Stand-up and sit-down chair test (number of times standing up from the chair within 30 s), ALT Arm lifting test (30 s dumb bell of 5 pounds or 2.3Kg lifting test), ANCOV, Analysis of Covariance, ANOVA Analysis of Variance, * *p* < 0.05

### Secondary outcomes

#### Stand-up and sit-down chair test (SPSDCT)

Table 2 shows the mean SPSDCT of two groups respectively at baseline and 12 weeks measurements we had scheduled. The intervention group showed an obvious increase in the numbers of SPSDCT, and the difference was significant (*p* = 0.000). The control group increased in the SPSDCT numbers, but the difference was not significant (*p* = 0.180). There were statistically significant differences among the two groups (*p* = 0.000).

#### Arm lifting test (ALT)

The difference for the intervention group was significant (*p* = 0.000), and there was no significant difference in the control group (*p* = 0.268). The changes of ALT in two groups showed a similar tendency. However, There were statistically significant differences between two groups (*p* = 0.017). The specific results and statistical analysis are shown in Table 2.

#### VO2max

The changes of VO2max in two groups showed a similar tendency (Table 2). The intervention group and the control group have a degree of increase after 12 weeks. The difference in the intervention group was significant (*p* = 0.027), but there was no significant difference in the control group (*p* = 0.056). There was no significant difference between the two groups (*p* = 0.149).

## Discussion

The aim of this study was to estimate the effects of the CEIBISMS on the QOL, muscle strength and cardiorespiratory capacity in postoperative BC patients with BC in China. In the present study, according to the changes of SF-36, SPSDCT, ALT and VO2max at baseline as well as at 12 weeks after the combined exercise intervention, we demonstrated that the CEIBISMS resulted in a statistically significant improvement in many subdomains of the SF-36, SPSDCT and ALT, which in turn was associated with improvements in QOL, muscle strength. The intervention group had significant improvement in VO2max compared with the control group. The CEIBISMS had a effect of improving cardiorespiratory in postoperative patients with BC in China.

The SF-36 can be applied to assess the QOL of BC patients [26–29]. Previous studies have shown that physical exercise management can significantly improve the QOL of BC patients [13, 30, 31]. However, few clinical trials have verified whether diversified exercise interventions have an increased beneficial effect on the QOL of BC patients. According to our research results, after 12 weeks of exercise intervention, the indexes of “vitality”, “mental health”, and “reported health transition” in SF-36 improved in the intervention group compared with the control group, with significant differences between the groups. The enhancement in vitality suggested that internet-based diversified exercise interventions can improve the subjective perception of BC patients toward vitality and fatigue. Since the sense of fatigue is common among postoperative survivors of BC [32, 33], the improvement of vitality has important implications for the enhancement of their QOL [34–36]. Patients with BC usually have psychosocial problems due to illness and disability caused by the surgery [37, 38]. The results of the research on the index of mental health indicated that internet-based diversified exercise interventions can improve the psychological aspects of BC patients. The results of reported health transition revealed that the overall health status of the subjects improved after 12 weeks of intervention. The indexes of “role-physical” and “general health” in the SF-36, after 12 weeks of exercise intervention, although without significant difference between the groups, showed an increase within the intervention group. The results of the role-physical indicated that BC survivors in the intervention group achieved some improvement in their functional limitations caused by their physiological health problems. The results of general health showed that BC survivors in the intervention group improved their own health status and its development trends. In conclusion, judging from the results of the study, 12-week of the CEIBISMS improved the QOL of postoperative BC patients.

SPSDCT and ALT can reflect the muscle strength of lower and upper extremities. After 12 weeks of diversified exercise interventions, the intervention group showed significant improvement compared with the control group. These improvements illustrated that the CEIBISMS can promote the development of muscle strength in BC patients. When arm curl test was performed, only the non-affected arm was tested in consideration that the subjects may refuse to complete the test for fear of causing or aggravating lymphedema of the affected limb. However, when the intervention was conducted, the bilateral arms were rehabilitated together. Therefore, the enhancement of the strength of the non-affected arm muscle strength can be used to speculate the improvement of muscle strength in the affected arm. In addition, there were no side effects that caused or aggravated lymphedema during the intervention, which also confirmed the conclusions of previous studies that resistance training does not cause or aggravate lymphedema in the affected side of BC survivors [39–43].

In previous studies, approximately 85% of women diagnosed with BC for the first time were over the age of 50, so the majority of survivors of BC were older women [44]. The improvement of muscle strength can promote balance control ability [45, 46] effectively preventing falls. Falls among the elderly are a major risk. Among the many factors causing falls of the elderly, muscle weakness is the main one [47]. In addition to causing falls, muscle weakness can trigger other diseases, especially the limitation of physical function or even loss of self-care ability [48–50]. Therefore muscle strength is of essential importance for postoperative survivors of BC. As suggested in the research results, internet-based diversified exercise interventions can reduce the risk of falling and future diseases of BC survivors by improving muscle strength.

It has been reported that about 60% BC survivors suffered from significant decrease in muscle strength of their upper limbs after surgery, seriously affecting the daily life of BC patients, thereby reducing their QOL [51]. Therefore, the improvement of muscle strength may have a positive effect on improving their QOL [52, 53]. As manifested in the research results, the improvement of QOL of BC survivors, to some extent, can be attributed to the improvement of muscle strength of the upper limbs.

Cardiorespiratory capacity was assessed by estimating the VO2max of each participant [54]. In previous studies it was reported that the improvement of cardiorespiratory endurance is positively correlated with the reduction of mortality in cancer patients [55, 56], showing the importance of cardiorespiratory for BC patients [57, 58]. The results of this study revealed that, after 12 weeks of intervention, although the difference regarding the VO2max between the intervention group and the control group showed no statistical significance, there was a trend of improvement in the intervention group through comparison within the group. Our training method for cardiorespiratory endurance was to let the subjects walk within a specified time to finish certain number of steps, but this exercise method, compared with cycle ergometer exercise [59, 60], had a difficulty in controlling exercise intensity, which may affect further improvement of cardiorespiratory endurance of the subjects. After a short-term intervention of 12 weeks, there was a trend of improvement in cardiorespiratory endurance of subjects in the intervention group. If medical supervision is further strengthened in cardiorespiratory endurance training and the intervention period is extended, better outcome would be expected.

No follow-up after study was the limitation of this study. In addition, the study period may have been too short to see the full benefits of the CEIBISMS on the improvement of QOL and cardiorespiratory capacity. We should do follow-up after the study and increase the intervention time in the future study.

## Conclusion

According to the results of this study, the CEIBISMS had rehabilitative effects on QOL and muscle strength in postoperative patients with BC in China. However, a longer duration of intervention trial is needed to confirm the effects of the CEIBISMS on BC in the future.

## Data Availability

The datasets used and analyzed during the current study are available from the corresponding author on reasonable request.

## Abbreviations

ALT: Arm lifting test (30 s dumbbell of 5 pounds or 2.3 Kg lifting test)
ANCOV: Analysis of Covariance
BC: Breast cancer
CEIBISMS: Combined Exercise Intervention Based on Internet and Social Media Software
CONSORT: Consolidated Standards of Reporting Trials
QOL: Quality of life
SF-36: the 36-item Short Form Health Survey
SPSDCT: Stand-up and sit-down chair test (number of times standing up from the chair within 30 s)

## References

[1] Breast cancer: prevention and control [http://www.who.int/topics/cancer/breastcancer/zh/]. Accessed 16 Feb 2018.

[2] Gokal K, Wallis D, Ahmed S, Boiangiu I, Kancherla K, Munir F (2016). Effects of a self-managed home-based walking intervention on psychosocial health outcomes for breast cancer patients receiving chemotherapy: a randomised controlled trial. Support Care Cancer.

[3] Qiu H, Ren W, Yang Y, Zhu X, Mao G, Mao S, Lin Y, Shen S, Li C, Shi H (2018). Effects of cognitive behavioral therapy for depression on improving insomnia and quality of life in Chinese women with breast cancer: results of a randomized, controlled, multicenter trial. Neuropsychiatr Dis Treat.

[4] Nakamura Y, Kitamura Y, Sumiyoshi Y, Naito N, Kan S, Ushio S, Miyazaki I, Asanuma M, Sendo T (2018). Involvement of 5-HT2A receptor hyperfunction in the anxiety-like behavior induced by doxorubicin and cyclophosphamide combination treatment in rats. J Pharmacol Sci.

[5] Leach HJ, Danyluk JM, Nishimura KC, Culos-Reed SN (2015). Evaluation of a community-based exercise program for breast Cancer patients undergoing treatment. Cancer Nurs.

[6] Dieli-Conwright CM, Courneya KS, Demark-Wahnefried W, Sami N, Lee K, Sweeney FC, Stewart C, Buchanan TA, Spicer D, Tripathy D (2018). Aerobic and resistance exercise improves physical fitness, bone health, and quality of life in overweight and obese breast cancer survivors: a randomized controlled trial. Breast Cancer Res.

[7] Ng AV, Cybulski AN, Engel AA, Papanek PE, Sheffer MA, Waltke LJ, Tjoe JA (2017). Triathlon training for women breast cancer survivors: feasibility and initial efficacy. Support Care Cancer.

[8] Stan DL, Croghan KA, Croghan IT, Jenkins SM, Sutherland SJ, Cheville AL, Pruthi S (2016). Randomized pilot trial of yoga versus strengthening exercises in breast cancer survivors with cancer-related fatigue. Support Care Cancer.

[9] Baruth M, Wilcox S, Der Ananian C, Heiney S (2015). Effects of home-based walking on quality of life and fatigue outcomes in early stage breast Cancer survivors: a 12-week pilot study. J Phys Act Health.

[10] Kim TH, Chang JS, Kong ID (2017). Effects of exercise training on physical fitness and biomarker levels in breast Cancer survivors. J Lifestyle Med.

[11] Dos Santos WDN, Gentil P, de Moraes RF, Ferreira Junior JB, Campos MH, de Lira CAB, Freitas Junior R, Bottaro M, Vieira CA (2017). Chronic effects of resistance training in breast Cancer survivors. Biomed Res Int.

[12] Knobf MT, Jeon S, Smith B, Harris L, Thompson S, Stacy MR, Insogna K, Sinusas AJ (2017). The Yale fitness intervention trial in female cancer survivors: cardiovascular and physiological outcomes. Heart Lung.

[13] Uhm KE, Yoo JS, Chung SH, Lee JD, Lee I, Kim JI, Lee SK, Nam SJ, Park YH, Lee JY, Hwang JH (2017). Effects of exercise intervention in breast cancer patients: is mobile health (mHealth) with pedometer more effective than conventional program using brochure?. Breast Cancer Res Treat.

[14] McCarroll ML, Armbruster S, Pohle-Krauza RJ, Lyzen AM, Min S, Nash DW, Roulette GD, Andrews SJ, von Gruenigen VE (2015). Feasibility of a lifestyle intervention for overweight/obese endometrial and breast cancer survivors using an interactive mobile application. Gynecol Oncol.

[15] Coughlin SS, Whitehead M, Sheats JQ, Mastromonico J, Smith S (2016). A review of smartphone applications for promoting physical activity. Jacobs J Community Med.

[16] Myers JS, Mitchell M, Krigel S, Steinhoff A, Boyce-White A, Van Goethem K, Valla M, Dai J, He J, Liu W (2018). Qigong intervention for breast cancer survivors with complaints of decreased cognitive function. Support Care Cancer.

[17] Liu P, You J, Loo WTY, Sun Y, He Y, Sit H, Jia L, Wong M, Xia Z, Zheng X (2017). The efficacy of Guolin-qigong on the body-mind health of Chinese women with breast cancer: a randomized controlled trial. Qual Life Res.

[18] Jong MC, Boers I, Schouten van der Velden AP, Meij SV, Goker E, Timmer-Bonte A, van Wietmarschen HA (2018). A randomized study of yoga for fatigue and quality of life in women with breast Cancer undergoing (neo) adjuvant chemotherapy. J Altern Complement Med.

[19] Graetz I, McKillop CN, Stepanski E, Vidal GA, Anderson JN, Schwartzberg LS (2018). Use of a web-based app to improve breast cancer symptom management and adherence for aromatase inhibitors: a randomized controlled feasibility trial. J Cancer Surviv.

[20] Early and locally advanced breast cancer: Diagnosis and management [https://www.nice.org.uk/guidance/ng101]. Accessed 20 Oct 2018.

[21] American College of Sports Medicine, Riebe D, Ehrman JK, Liguori G, Magal M. ACSM's guidelines for exercise testing and prescription. Tenth edition. edn. Philadelphia: Wolters Kluwer;2018.

[22] Serra MC, Ryan AS, Ortmeyer HK, Addison O, Goldberg AP (2018). Resistance training reduces inflammation and fatigue and improves physical function in older breast cancer survivors. Menopause.

[23] Dolan LB, Barry D, Petrella T, Davey L, Minnes A, Yantzi A, Marzolini S, Oh P (2018). The cardiac rehabilitation model improves fitness, quality of life, and depression in breast Cancer survivors. J Cardiopulm Rehabil Prev.

[24] Xiaosheng D, Xiangren Y, Shuyuan H, Dezong G, Mengyao C, Meng D (2018). The effects of combined exercise intervention based on internet and social media software for postoperative patients with breast cancer: study protocol for a randomized controlled trial. Trials.

[25] Baker TB, Gustafson DH, Shaw B, Hawkins R, Pingree S, Roberts L, Strecher V (2010). Relevance of CONSORT reporting criteria for research on eHealth interventions. Patient Educ Couns.

[26] Pakiz B, Ganz PA, Sedjo RL, Flatt SW, Demark-Wahnefried W, Liu JX, Wolin KY, Rock CL (2016). Correlates of quality of life in overweight or obese breast cancer survivors at enrollment into a weight loss trial. Psycho-Oncology.

[27] Winters-Stone KM, Medysky ME, Savin MA (2018). Patient-reported and objectively measured physical function in older breast cancer survivors and cancer-free controls. J Geriatr Oncol.

[28] Goyal NG, Ip EH, Salsman JM, Avis NE (2018). Spirituality and physical health status: a longitudinal examination of reciprocal effects in breast cancer survivors. Support Care Cancer.

[29] Sleight AG, Lyons KD, Vigen C, Macdonald H, Clark F (2018). The association of health-related quality of life with unmet supportive care needs and sociodemographic factors in low-income Latina breast cancer survivors: a single-Centre pilot study. Disabil Rehabil.

[30] Rincon E, Monteiro-Guerra F, Rivera-Romero O, Dorronzoro-Zubiete E, Sanchez-Bocanegra CL, Gabarron E (2017). Mobile phone apps for quality of life and well-being assessment in breast and prostate Cancer patients: systematic review. JMIR Mhealth Uhealth.

[31] Pope ZC, Zeng N, Zhang R, Lee HY, Gao Z (2018). Effectiveness of combined smartwatch and social media intervention on breast Cancer survivor health outcomes: a 10-week pilot randomized trial. J Clin Med.

[32] Reinertsen KV, Engebraaten O, Loge JH, Cvancarova M, Naume B, Wist E, Edvardsen H, Wille E, Bjoro T, Kiserud CE (2017). Fatigue during and after breast Cancer therapy-a prospective study. J Pain Symptom Manag.

[33] Nieboer P, Buijs C, Rodenhuis S, Seynaeve C, Beex LV, van der Wall E, Richel DJ, Nooij MA, Voest EE, Hupperets P (2005). Fatigue and relating factors in high-risk breast cancer patients treated with adjuvant standard or high-dose chemotherapy: a longitudinal study. J Clin Oncol.

[34] Ahmed AE, Almuzaini AS, Alsadhan MA, Alharbi AG, Almuzaini HS, Ali YZ, Jazieh AR (2018). Health-related predictors of quality of life in Cancer patients in Saudi Arabia. J Cancer Educ.

[35] Pisu M, Demark-Wahnefried W, Kenzik KM, Oster RA, Lin CP, Manne S, Alvarez R, Martin MY (2017). A dance intervention for cancer survivors and their partners (RHYTHM). J Cancer Surviv.

[36] Speed-Andrews AE, Stevinson C, Belanger LJ, Mirus JJ, Courneya KS (2010). Pilot evaluation of an Iyengar yoga program for breast cancer survivors. Cancer Nurs.

[37] Matousek RH, Pruessner JC, Dobkin PL (2011). Changes in the cortisol awakening response (CAR) following participation in mindfulness-based stress reduction in women who completed treatment for breast cancer. Complement Ther Clin Pract.

[38] Abbasi B, Mirzakhany N, Angooti Oshnari L, Irani A, Hosseinzadeh S, Tabatabaei SM, Haghighat S (2018). The effect of relaxation techniques on edema, anxiety and depression in post-mastectomy lymphedema patients undergoing comprehensive decongestive therapy: a clinical trial. PLoS One.

[39] Baumann FT, Reike A, Reimer V, Schumann M, Hallek M, Taaffe DR, Newton RU, Galvao DA (2018). Effects of physical exercise on breast cancer-related secondary lymphedema: a systematic review. Breast Cancer Res Treat.

[40] de Oliveira MMF, Gurgel MSC, Amorim BJ, Ramos CD, Derchain S, Furlan-Santos N, dos Santos CC, Sarian LO (2018). Long term effects of manual lymphatic drainage and active exercises on physical morbidities, lymphoscintigraphy parameters and lymphedema formation in patients operated due to breast cancer: a clinical trial. PLoS One.

[41] Ammitzboll G, Lanng C, Kroman N, Zerahn B, Hyldegaard O, Andersen KK, Johansen C, Dalton SO (2017). Progressive strength training to prevent LYmphoedema in the first year after breast CAncer - the LYCA feasibility study. Acta Oncol.

[42] Nelson NL (2016). Breast Cancer-related lymphedema and resistance exercise: a systematic review. J Strength Cond Res.

[43] Ahmed RL, Thomas W, Yee D, Schmitz KH (2006). Randomized controlled trial of weight training and lymphedema in breast cancer survivors. J Clin Oncol.

[44] Winters-Stone KM, Dobek J, Bennett JA, Nail LM, Leo MC, Schwartz A (2012). The effect of resistance training on muscle strength and physical function in older, postmenopausal breast cancer survivors: a randomized controlled trial. J Cancer Surviv.

[45] Orr R (2010). Contribution of muscle weakness to postural instability in the elderly. A systematic review. Eur J Phys Rehabil Med.

[46] Twiss JJ, Waltman NL, Berg K, Ott CD, Gross GJ, Lindsey AM (2009). An exercise intervention for breast cancer survivors with bone loss. J Nurs Scholarsh.

[47] Guideline for the prevention of falls in older persons (2001). American Geriatrics Society, British geriatrics society, and American Academy of Orthopaedic surgeons panel on falls prevention. J Am Geriatr Soc.

[48] Bennett JA, Winters-Stone K, Nail L (2006). Conceptualizing and measuring physical functioning in cancer survivorship studies. Oncol Nurs Forum.

[49] Rantanen T (2003). Muscle strength, disability and mortality. Scand J Med Sci Sports.

[50] Fried LP, Bandeen-Roche K, Chaves PH, Johnson BA (2000). Preclinical mobility disability predicts incident mobility disability in older women. J Gerontol A Biol Sci Med Sci.

[51] Bicego DBK, Ruddick M, Storey D, Wong C, Harris SR (2016). Exercise for women with or at risk for breast cancer-related lymphedema. Phys Ther.

[52] Schulz SVW, Laszlo R, Otto S, Prokopchuk D, Schumann U, Ebner F, Huober J, Steinacker JM (2018). Feasibility and effects of a combined adjuvant high-intensity interval/strength training in breast cancer patients: a single-center pilot study. Disabil Rehabil.

[53] Schmidt T, Berner J, Jonat W, Weisser B, Rocken C, van Mackelenbergh M, Mundhenke C (2017). Influence of arm crank ergometry on development of lymphedema in breast cancer patients after axillary dissection: a randomized controlled trail. J Rehabil Med.

[54] oraya Casla SLp-T, Yolanda Jerez, Iva’n Marquez-Rodas, Daniel A (2015). Galva˜o supervised physical exercise improves VO2max, quality of life, and health in early stage breast cancer patients: a randomized controlled trial. Breast Cancer Res Treat.

[55] Betof AS, Dewhirst MW, Jones LW (2013). Effects and potential mechanisms of exercise training on cancer progression: a translational perspective. Brain Behav Immun.

[56] Warburton DE, Nicol CW, Bredin SS (2006). Health benefits of physical activity: the evidence. CMAJ.

[57] Karlsen T, Aamot IL, Haykowsky M, Rognmo O (2017). High intensity interval training for maximizing health outcomes. Prog Cardiovasc Dis.

[58] Tolentino GP, Battaglini CL, Araujo SS, Otano AS, Conde DM, Evans ES, de Oliveira RJ (2010). Cardiorespiratory fitness and quality-of-life analysis posttreatment in breast cancer survivors. J Psychosoc Oncol.

[59] Evans ES, Hackney AC, McMurray RG, Randell SH, Muss HB, Deal AM, Battaglini CL (2015). Impact of acute intermittent exercise on natural killer cells in breast Cancer survivors. Integr Cancer Ther.

[60] Dolan LB, Lane K, McKenzie DC (2012). Optimal mode for maximal aerobic exercise testing in breast cancer survivors. Integr Cancer Ther.

